# Advances in Fibrin-Based Materials in Wound Repair: A Review

**DOI:** 10.3390/molecules27144504

**Published:** 2022-07-14

**Authors:** Ilker S. Bayer

**Affiliations:** Smart Materials, Istituto Italiano di Tecnologia, Via Morego 30, 16163 Genova, Italy; ilker.bayer@iit.it

**Keywords:** fibrin, fibrinogen, wound healing, drug release, protein, nanofibers

## Abstract

The first bioprocess that occurs in response to wounding is the deterrence of local hemorrhage. This is accomplished by platelet aggregation and initiation of the hemostasis cascade. The resulting blood clot immediately enables the cessation of bleeding and then functions as a provisional matrix for wound healing, which begins a few days after injury. Here, fibrinogen and fibrin fibers are the key players, because they literally serve as scaffolds for tissue regeneration and promote the migration of cells, as well as the ingrowth of tissues. Fibrin is also an important modulator of healing and a host defense system against microbes that effectively maintains incoming leukocytes and acts as reservoir for growth factors. This review presents recent advances in the understanding and applications of fibrin and fibrin-fiber-incorporated biomedical materials applied to wound healing and subsequent tissue repair. It also discusses how fibrin-based materials function through several wound healing stages including physical barrier formation, the entrapment of bacteria, drug and cell delivery, and eventual degradation. Pure fibrin is not mechanically strong and stable enough to act as a singular wound repair material. To alleviate this problem, this paper will demonstrate recent advances in the modification of fibrin with next-generation materials exhibiting enhanced stability and medical efficacy, along with a detailed look at the mechanical properties of fibrin and fibrin-laden materials. Specifically, fibrin-based nanocomposites and their role in wound repair, sustained drug release, cell delivery to wound sites, skin reconstruction, and biomedical applications of drug-loaded fibrin-based materials will be demonstrated and discussed.

## 1. Fibrin(ogen) Structure and Function

**Figure 1:**
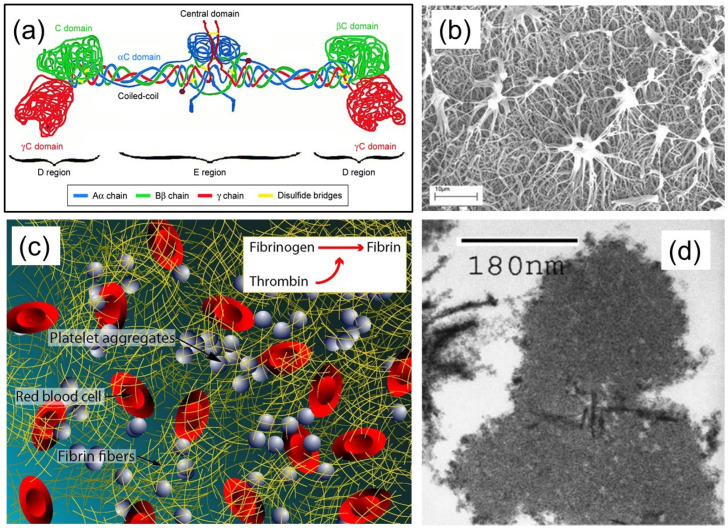
(**a**) Fibrinogen structure. Aα chains are shown in blue, Bβ chains are shown in green, and γ chains are shown in red. Disulfide bridges stabilizing the coiled-coil regions are shown in yellow. Reprinted/adapted with permission from Ref. [5], 2017, DovePress. (**b**) SEM image of fibrin clots formed using high and low thrombin concentrations after 10 min of lysis after the addition of tissue-type plasminogen activator (tPA) to the surface of the clot. Reprinted/adapted with permission from Ref. [6], 2011, American Society of Hematology. (**c**) Image of how fibrin fibers from fibrinogen act as glue and a scaffold for the grouping of red blood cells, platelets, and other plasma proteins within a fibrin clot. Reprinted/adapted with permission from Ref. [7], 2017, Elsevier. (**d**) TEM image (90,000×) of the cross-section of a fibrin fiber. Reprinted/adapted with permission from Ref. [8], 2004, Elsevier.

Fibrinogen and fibrin play intersecting roles in several functions such as regulating blood clotting, fibrinolysis, cellular and matrix interactions, the inflammatory response, wound healing, and neoplasia (the process of abnormal and excessive tissue growth) [1]. These biological processes are regulated by interactive sites on fibrin(ogen), some of which are masked or not present on fibrinogen, and other sites can evolve during fibrin formation or during fibrinogen–surface chemical interactions. The structural features of fibrin(ogen) can be summarized as fibrin polymerization and cross-linking, leading to many biological functions such as binding to thrombin, fibrinolysis, the regulation of factor XIII (coagulation protein) activity, growth factor binding, and interactions with cells including platelets, leukocytes, fibroblasts, and endothelial cells [2]. More specifically, fibrin is the major protein component of blood clots and is fashioned from fibrinogen, a glycoprotein that circulates inertly in the blood stream. Fibrinogen is composed of six polypeptide chains (Aα, Bβ, γ)2, held together by disulfide bonds in a molecular structure with bilateral symmetry, as depicted in its simplest form in Figure 1a. The molecular structure is complex and is constructed from three main structural regions. The first section is a central region (E) which contains fibrinopeptides A and B and the amino acid termini of all six polypeptide chains. This is followed by two distal regions (D) connected to the E region by two α-helical coiled segments, and with D regions having the carboxyl termini of the Bβ and γ chains, and those of the Aα chain, which extend out, forming flexible αC-domains, each terminating with a globular domain [3,4].

The transformation of fibrinogen to fibrin is prompted by the thrombin-catalyzed release of small peptides from the amino-terminal segments of the a- and b-chains, denoted as fibrinopeptides A and B, respectively, as shown above. Due to the removal of the peptides, the parent molecules polymerize spontaneously. The polymerization process itself can be divided into a number of individual steps, beginning with the removal of one or both of each kind of fibrinopeptide. Different polymerization paths may result, depending on the relative release rates of the A and B fibrinopeptides. The paths are also dependent on the media or the environment, including the pH, ionic strength, temperature, and the presence or absence of other small molecules and proteins [2,6]. In fact, fibrin polymer is the final product of the enzymatic cascade of blood clotting. In parallel, fibrinolysis is a process that prevents blood clots from growing and becoming problematic. Primary fibrinolysis is a normal body process, whereas secondary fibrinolysis is the breakdown of clots due to a medicine, a medical disorder, or some other cause. In fibrinolysis, a fibrin clot, the product of coagulation, is broken down (i.e., lysis). Its main enzyme plasmin cuts the fibrin mesh at various places, leading to the production of circulating fragments that are cleared by other proteases or by the kidney and liver. Plasmin is produced in an inactive form, plasminogen, in the liver. Although plasminogen cannot cleave fibrin, it has high affinity for it, and is incorporated into the clot when the clot starts forming. Tissue plasminogen activator (tPA) and urokinase are the agents that convert plasminogen to the active plasmin, thus allowing fibrinolysis to occur. The scanning electron microscopy image in Figure 1b demonstrates an example of the enzymatic fibrin lysis (break down) process with tPA. The aggregates eventually break free from the fiber network and mix into the bloodstream.

In vivo formation of the fibrin polymer network, along with platelet adhesion and aggregation, are key events in the elimination of bleeding at the site of injury (hemostasis), as well as in pathological vascular occlusion (thrombosis). More simply, the polymerized fibrin, together with platelets, forms a hemostatic plug or clot over a wound site. When the lining of a blood vessel is broken, platelets are attracted, forming a platelet group, as seen in Figure 1c. These platelets have thrombin receptors on their surfaces that bind serum thrombin molecules which, in turn, convert soluble fibrinogen in the serum into fibrin at the wound site. Researchers have also been very interested in the structure of individual fibrin fibers. Examining the cross-sectional area of individual fibrin fiber in transmission electron microscopy (TEM) indicates that the single fiber is an arrangement of densely packed irregular shapes of different sizes containing a few irregular voids or channels (see Figure 1d). Notably, to take TEM images, the samples need to be under vacuum conditions; these conditions can cause severe dehydration and subsequent collapse of the fibers.

**Figure 2:**
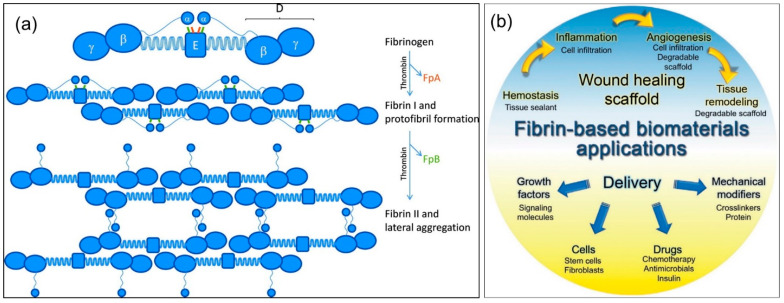
(**a**) Fibrinogen to fibrin formation. The α-chain termini fold back on the coiled-coil and interact with the E-region. Fibrin formation involves the cleavage of FpA (orange) by thrombin (fibrin I), which polymerizes into protofibrils. Subsequently, FpB (green) is cleaved by thrombin (fibrin II). FpB cleavage is associated with release of the α-domains, which interact for lateral aggregation. Reprinted/adapted with permission from Ref. [9], 2011, American Heart Association, Inc. (**b**) The applications of fibrin-based biomaterials are very diverse and mirror the biological roles of fibrin in wound repair. Fibrin-based biomaterials can be applied as a wound healing scaffold to promote hemostasis immediately following injury, and then provide the structure for subsequent inflammatory cell infiltration, angiogenesis, and long-term tissue remodeling. Reprinted/adapted with permission from Ref. [10], 2018, Elsevier.

As mentioned above, fibrin polymerization encompasses a number of successive reactions, each affecting the ultimate structure and properties of the fibrin framework. Figure 2a schematically demonstrates the polymerization process, starting from fibrinogen. These properties govern the development and outcomes of various diseases, such as heart attack, ischemic stroke, cancers, trauma, surgical and obstetrical complications, hereditary and acquired coagulopathies, and thrombocytopathies. Understanding the pathogenesis of such disorders, along with better insight into the molecular mechanisms of fibrin formation, allows us to develop new diagnostic tools and therapeutic approaches, including wound repair [6].

In parallel, notable recent studies also discovered synthetic fibrin cross-linking polymers for modulating clot properties and inducing hemostasis [11]. Extensive blood loss is a principal cause of death after trauma, and many intravenously delivered clotting agents in use today are expensive, require special storage, have limited shelf life, and carry risks of immunogenicity. Chan et al. [11] demonstrated that a commercial synthetic, bioinspired polymer called PolySTAT could stop the bleeding after trauma and re-established hemostasis. Their research has showed that in healthy animals, the polymer circulated throughout the body, and remained harmless and passive. Upon encountering a blood clot at a site of vascular injury, the polymer initiated the cross-linking of fibrin into a matrix, similar to the transglutaminase factor XIII, consolidating the clot and fortifying it against enzymes that are overactive in trauma. The authors acknowledged that further preclinical studies will be conducted in larger animal models to evaluate issues such as clotting function and the extent of organ injury in surviving animals [9,11]. These future studies should be supported by powerful modeling simulations on fibrin formation based on crystal structures of fibrinogen and fibrin fragments complexed with synthetic peptides [9,12].

The biomedical applications of fibrin-based biomaterials depicted in Figure 2b are very diverse and reflect the biological roles of fibrin in wound repair. Fibrin-based biomaterials can be designed as wound healing scaffolds to promote hemostasis immediately after injury, and they can provide the structure for subsequent inflammatory cell infiltration, angiogenesis, and long-term tissue remodeling [8,10,12,13,14,15]. This review focuses on various pre-clinical and clinical approaches developed for the regeneration of damaged tissues and the wound healing process using fibrin-based biomaterials. In particular, dimensionally modified fibrin materials (two- or three-dimensional scaffolds) or its phasic characteristics (injectable or implantable matrices) that have been fabricated to promote biological interactions are reviewed, and their effects on optimal tissue regeneration, wound healing, and functioning restoration are presented and discussed. The review also shows that depending on the wound severity and chronic nature such as various tissue defects or physiological environments, fibrin-based materials with proper drug loading and sustained release capacity can be used to design several therapeutic materials and techniques to improve the mechanical, physical, chemical, or biological properties of the wound healing process.

## 2. Properties of Fibrin Fibers

**Figure 3:**
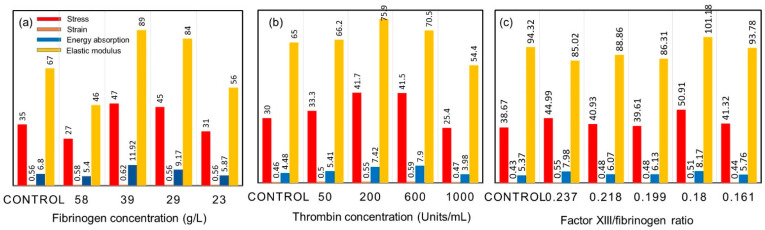
(**a**) Results of mechanical testing when the fibrinogen concentration of the glue is varied, keeping the thrombin (200 units/mL), aprotinin (3000 Klu/mL), and calcium (40 μmol/mL) levels constant. (**b**) Results of mechanical testing when the thrombin concentration of the glue is regulated, keeping the fibrinogen (39 g/L), aprotinin (3000 Klu/mL), and calcium (40 μmol/mL) levels constant. (**c**) Results of mechanical testing when the factor XIII/fibrinogen ratio is varied, keeping the thrombin (200 units/mL), aprotinin (3000 KIu/mL) and calcium (40 μmol/mL) levels constant. All measurements reported in the plots are the mean *p* < 0.01, difference from control; Mann–Whitney U test. Data were compiled from [23].

Fibrin is a viscoelastic polymer with both elastic and viscous properties. The elasticity (or stiffness) is attributed to reversible mechanical deformation, whereas viscosity (or plasticity) is due to irreversible deformation induced by shear or force. The elastic component of clots formed by fibrin polymerization is generally about one order of magnitude higher than the viscous component. Notably, the viscous component increases rapidly at higher rates of deformation. It has been reported that based on controlled creep experiments, in which continued changes in strain are measured over time after the application of stress, the stiffness of some clots does not change, indicating that fibrin is a “self-repairing” polymer. This property may be attributed to the existence of the knob–hole bonds holding the structure together being reversible [16,17]. A blood clot must develop the right degree of stiffness and plasticity to block the flow of blood, and yet be digestible or degradable by lytic enzymes to avoid the formation of a thrombus that can lead to heart attacks, strokes, or pulmonary emboli. Clearly, this unique property requires fibrin polymers to demonstrate singular mechanical properties that are still difficult to apprehend. As shown in Figure 1c, clots are made up of a three-dimensional network of fibrin fibers established by ligation with a transglutaminase, factor XIIIa. Researchers have recently developed new methodologies to measure the elastic moduli of individual fibrin fibers in fibrin clots with or without ligation, using optical tweezers for trapping beads attached to fibers that function as anchors to strain a fiber [18]. For instance, single fibers were bridged between the grooves in a straight line approximately perpendicular to the ridge edge of a specifically fabricated substrate, as shown in Figure 3a. The anchored fibers were then subjected to tension using the tip of an atomic force microscope (AFM) to determine the mechanical properties [19].

Using the results of such measurements, it is now possible to build realistic mechanical models of a blood clot, along with recently developed network modeling approaches. The model data could then be compared with whole clot measurements [20,21,22]. Tissue glues based on fibrin, for example, have many advantages over non-biological adhesives. These glues must be formulated such that they mechanically reinforce healing wounds. Studies focused on this effect have demonstrated that fibrin glues can be designed using standard dorsal skin incisions in animal models by varying the fibrinogen, thrombin, and factor XIII concentrations. Commercial fibrin glues are multicomponent materials; however, in general their formulations contain about 80 g/L fibrinogen, about 500 units/mL thrombin, and 50 units/mL factor XIII. Excised wounds have been mechanically tested, and the results indicated that glues which did not contain any factor XIII resulted in wounds with significantly increased stress, energy absorption, and elasticity values [23].

Figure 3a indicates that variation in the fibrinogen concentration of the glue within the range of 23 g/L to 58 g/L alters the mechanical properties of glued wounds in a rat skin model after 8 days as compared with control wounds. Wounds treated with a glue with a fibrinogen concentration of 23 g/L did not differ much from control wounds, although they were slightly more elastic. With fibrinogen concentrations of 29 g/L and 39 g/L, the wounds were stronger (higher stress and energy values), but more rigid. Wounds closed with a glue with a fibrinogen concentration of 58 g/L exhibited lower stress and elasticity values than control wounds, although the strain and energy absorption results were not significantly different from those of control wounds. Mechanical testing of wounds closed with a glue with varying thrombin concentrations showed a similar pattern to the above results (Figure 3b). Wounds treated with a glue of a thrombin concentration of 50 units/mL showed no difference from control wounds. Wounds treated with a glue with thrombin concentrations of 200 units/mL and 600 units/mL resulted in wounds exhibiting significantly higher stress, strain, energy absorption, and elasticity values from control wounds. A thrombin concentration of 1000 units/mL in the glue resulted in considerably more elastic wounds with lower stress values. Alterations in the factor XIII/fibrinogen ratio of the glue did not modify the mechanical properties necessary to rupture the wound when compared with the control wound cross-sectional area (Figure 3c). Additional varying concentrations of exogenous factor XI11 to the glue did not influence the mechanical properties of the glued and control wounds. For the latter two experiments involving factor XIII, a fibrinogen preparation deficient in factor XIII was used. Microscopy of the wound sections stained for fibrin showed no fibrin in the wounds after 8 days. Normally, in vivo clots and thrombin contain erythrocytes, or red blood cells (RBCs), at this stage. Notable studies were conducted to determine the effects of RBCs on fibrin clot mechanical properties by comparing plasma clots without RBCs to those prepared with low (2 vol.%), intermediate (5–10 vol.%), or high (≥20 vol.%) numbers of RBCs. It was reported that low RBC concentrations had little effect on clot structure. Intermediate RBC concentrations triggered heterogeneity in the fiber network, with pockets of densely packed fibers together with regions with few fibers. With high levels of RBCs, fibers were arranged more uniformly but loosely around the cells.

Moreover, the ratio of viscous modulus (G″) to elastic modulus (G′) increased significantly over that of a clot without any RBCs. These results could be used for better understanding in vivo clots and thrombi [24]. Additionally, a recent study by Xia et al. [25] demonstrated that during clotting, activated platelets released Zn^2+^ ions that surprisingly reduced fibrin gel stiffness, although it induced bundling that toughened the network. As such, it is safe to state that the mechanics of fibrin networks are dependent on the formed microscopic structure and can lead to better understanding of the physical mechanisms leading to defective or excessive clot stiffness during wound healing process.

**Figure 4:**
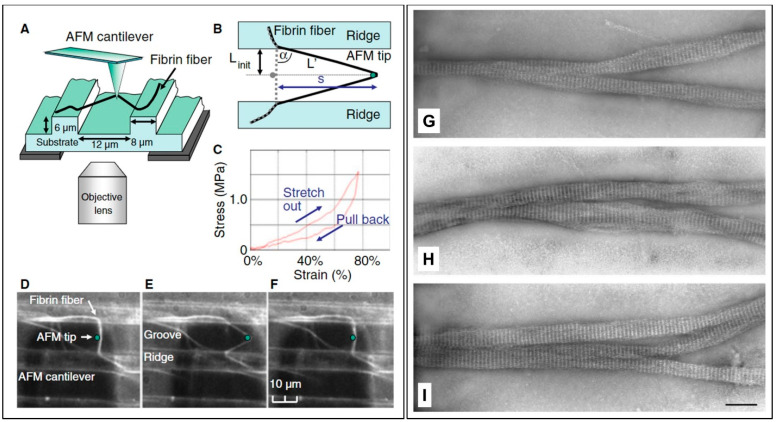
(**A**) Schematic of the atomic force microscope (AFM) sitting on top of the inverted optical microscope. (**B**) Top view of the stretched fiber. The initial and stretched states are in dotted gray and solid black, respectively. (**C**) Typical fibrin fiber stress–strain curve. (**D**–**F**) Fluorescence microscopy film stills of a stretching experiment. The fiber is anchored on two ridges (brighter, horizontal, 8 μm wide bars) and suspended over a groove (darker, horizontal, 12 μm wide bars); the AFM cantilever appears as a 35 μm wide, dark rectangle; the AFM tip is indicated as a green dot. Reprinted/adapted with permission from Ref. [19], 2010, International Society on Thrombosis and Haemostasis. (**G–I**) TEM images of negatively contrasted fibrin fibers showing the substructure of branch points. Most branch points consist of three fiber segments of nearly equal diameters that join at a small acute angle with band patterns aligned. The band pattern with a repeat of 22.5 nm is characteristic of fibrin. Scale bar is 0.2 microns. Reprinted/adapted with permission from Ref. [26], 2004, Elsevier.

From these studies, it is evident that the mechanical properties of fibrin are essential to the physiology of blood clotting and are important for understanding and eventually preventing and treating bleeding wounds. For instance, not only the fibrin fiber size, but the branching of fibrin fibers in clot networks directly affects wound closure and healing [26,27]. Experiments have focused on selecting single fibers bridging the grooves in a straight line almost perpendicular to the ridge edge. As such, most fibers are firmly anchored on the ridges of the striated substrate, even at extreme fiber extensions, excluding fibers that have slipped on the ridges. Such an experimental design yields a well-defined geometry to determine the mechanical properties of fibrin fibers (Figure 4A,B). For instance, a typical stress–strain curve of a fibrin fiber stretched to 1.75 times its initial length is shown in Figure 4C. There is hysteresis, which means that energy (the area under the curves) is lost in this stretching cycle. However, although energy is lost, there is no permanent elongation in the fiber. A typical film still of a fibrin fiber stretching experiment under the AFM tip is shown in Figure 4D,F. More details and the videos are presented in the study by Liu et al. [19]. Quantitative studies on clot structure demonstrated that nearly all branch points in clots are made up of three fibers at a junction, as seen in the TEM images of Figure 3b. Inspection of the stain patterns on the fibers in Figure 4G–I show that negatively contrasted fibrin reveals the substructure of the fibers, because the stain distribution is directly related to protein density. Regions of high protein density are less stained, making them appear bright, whereas regions of lower protein density are penetrated by the microscope staining agent, making them appear darker, such that fibrin fibers have a 22.5 nm periodicity with a typical band pattern (Figure 4G–I). According to Liu et al. [28], fibrin fibers can be strained 180% (2.8-fold extension) without sustaining permanent extension, and they can be strained up to 525% (average 330%) before rupturing. They claimed that these observations were the largest extensibility observed for any protein fibers. Their data implied that fibrin monomers must be able to undergo sizeable, reversible structural changes, and that deformations in clots could be accommodated by individual fiber stretching.

## 3. Mechanics of Platelet–Fibrin Interactions and Wound Healing

Wound healing is an intricate process which involves three entangled stages that follow each other as hemostatic and inflammatory reactions, the proliferative stage and the subsequent remodeling and maturation phase. The wound healing process is rigorously controlled by various cytokines and growth factors that are secreted to the wound area [29]. Platelets are cells that play a fundamental role in wound healing, and they are the main basis for the growth factor complex that significantly contributes to natural wound healing. They spread toward the wound region very fast and start coagulation [30]. Platelets not only facilitate clot formation and stop local losses of lymph and blood, but also prompt angiogenesis in wound healing and by stimulating mesenchymal cells. They are also engaged in the production of more than 20 growth factors that are required for tissue regeneration. Some proteins such as thrombin achieve the secretion of these factors by platelet degranulation [31]. As mentioned earlier, fibrin deformation and interactions of fibrin with other blood components play critical roles in wound healing and reconstruction. For this purpose, in addition to key experimental observations, multiscale models are needed to describe fibrin mechanics, deformation, contraction, and the role of biomechanical interactions between platelets and fiber networks in blood clot stretching and contraction models should be investigated in detail. Several models have shown that local strain-stiffening and pairwise interactions of individual fibers contribute to the mechanical responses of fibrin networks. However, several open problems and challenges remain, such as studying microscale mechanisms the multiscale model development, calibration, and verification. A detailed review on this issue has recently been discussed by Pancaldi et al. [32].

Blood clot contraction (retraction) is determined by platelet-generated forces that are transmitted by the fibrin network. This network regulates clot shrinkage and the deformation of erythrocytes. More specifically, during blood clot contraction, microscopic platelets actively pull fibers to shrink the macroscale clot to less than 10% of its initial volume [33]. To study the mechanical nature of this process, Tutwiler et al. [7] developed a model that unites an active contractile motor element with passive viscoelastic elements. Clot contraction is poorly understood, even though its importance in thrombosis and wound healing is acknowledged. Their model envisaged how clot contraction occurs due to active contractile platelets that interact with a viscoelastic material, instead of referring to the poroelastic nature of fibrin. Furthermore, their work attempts to explain the observed dynamics of clot size, ultrastructure, and measured forces. Erythrocytes and fibrin are not mechanically active, but are present in series and parallel to active contractile cells. This mechanical interplay imposes compressive and tensile resistance, triggering an increased contractile force and a reduced extent of contraction in the presence of erythrocytes. Their model was also validated experimentally and may form the fundamental mechanical basis for understanding blood clot contractions.

**Figure 5:**
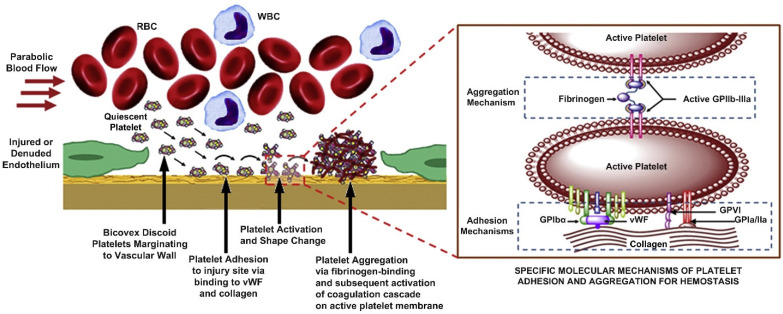
Physical and biological events in the hemostatic action of platelets. Reprinted/adapted with permission from Ref. [35], 2013, Elsevier.

To tackle the problems related to platelet depletion or dysfunction-induced inadequate wound healing, new materials are being developed that are synthetic and non-immunogenic. Platelet mimetic technologies to augment hemostatic responses for preventing deficient native platelet functionality are increasingly being reported. Already, some studies have developed synthetic platelet-like particles that can recapitulate the deformable platelet body and inherent fibrin specificity of native platelets, to enhance healing outcomes [34]. It was demonstrated that platelet-like particles mimicked activated platelet morphology and induced fibrin clot retraction. During clot retraction, natural platelets generate forces within the fibrin fiber network, stiffening it; therefore, these synthetic counterparts also stiffened the fibrin matrices, leading to increased cellular migration. Enhanced cell migration is known to improve hemostasis and wound healing outcomes within in vitro and in vivo models of wound healing [35] (see Figure 5).

The hemostatic function follows a number of steps such as migration to the vascular wall from bulk blood flow followed by stable adhesion to the injury site at the vascular wall through vascular matrix proteins and collagen and concomitant activation via the action of adenosine diphosphate (ADP) and thrombin. These stages are followed by aggregation at the adhesion site via fibrinogen-induced ligand–receptor activity, and co-localization of coagulation factors on the active platelet membrane, triggering the coagulation cascade, as shown in Figure 5 [36]. These platelet-rich fibrin systems were also shown to be quite effective in treating dental wounds, a subject reviewed by Naik et al. [37]. As mentioned before, in wound healing, the primary media are the fibrin network clot and the granulation tissue that is formed by newly deposited collagen. To represent these local microenvironments, biomimetic hydrogels made up of fibrin or collagen have been designed and used at the wound site. However, in order to properly recreate wound healing environments, it is also exceedingly important to study and understand the biomechanical and biophysical properties of such hydrogels in conjunction with the natural fibrin network [38].

## 4. Fibrin Nanocomposites in Wound Management

Fibrinogen, a natural soluble blood protein, can be converted to insoluble fibrin by active thrombin in the presence of other nanostructured natural matrices. These fibrin nanocomposites could be pivotal in hemostasis, tissue repair, and host defense against infections. In these nanocomposites, fibrin can be designed in different forms, each having a specific and unique structure–function profile. This variation in form and function provides nanocomposites with many opportunities to create new and improved customized healthcare solutions based on specific variants of human fibrinogen and fibrin. In this section, we review such studies in which fibrin was combined with other biomedical polymers such as chitosan, and nanocellulose-forming functional nanocomposites suitable for wound management [5,39,40]. Vedakumari et al. [41] produced chitosan–fibrin nanocomposites (CFNs). They investigated the anti-bacterial activity of CFNs against *Escherichia coli* and *Staphylococcus aureus*. As a drug delivery matrix, 71% of methotrexate (MTX) was entrapped in CFNs that sustained release rates for up to 96 h. They also investigated the role of CFN in wound healing by creating open excision wounds on albino rats. Topical application of CFN, once in 2 days, for up to 10 days, induced the complete healing of wounds on day 14, whereas it took about 1 week longer in control wounds. Histological and biochemical characterization indicated the increased synthesis of collagen with the active migration of fibroblasts and epithelial cells in CFN-treated wounds. In a similar study, Vedakumari et al. [42] used freeze-dry methods to fabricate novel quercetin-incorporated chitosan–fibrin [Q-CF] nanocomposites. The nanocomposites were antibacterial against *Escherichia coli* and *Staphylococcus aureus*. The in vivo experiments performed using albino rats showed that wounds treated with the nanocomposites had faster rates of healing than the test groups. Mohandas et al. [43] developed an injectable chitosan–fibrin (CF)-based nanocomposite hydrogel for angiogenic response (angiogenesis is the physiological process through which new blood vessels form from pre-existing vessels during the earlier stage of vasculogenesis). The hydrogel was composed of chitosan containing 3% weight fibrin. The authors assessed the cytocompatibility and angiogenic response of the CF hydrogel using human umbilical cord vein endothelial cells. In vitro tube formation and nitric oxide release assay indicated an improved angiogenic behavior of the nanocomposite hydrogel when compared with the control. Furthermore, angiogenesis was confirmed through an ex vivo aortic sprouting assay.

**Figure 6:**
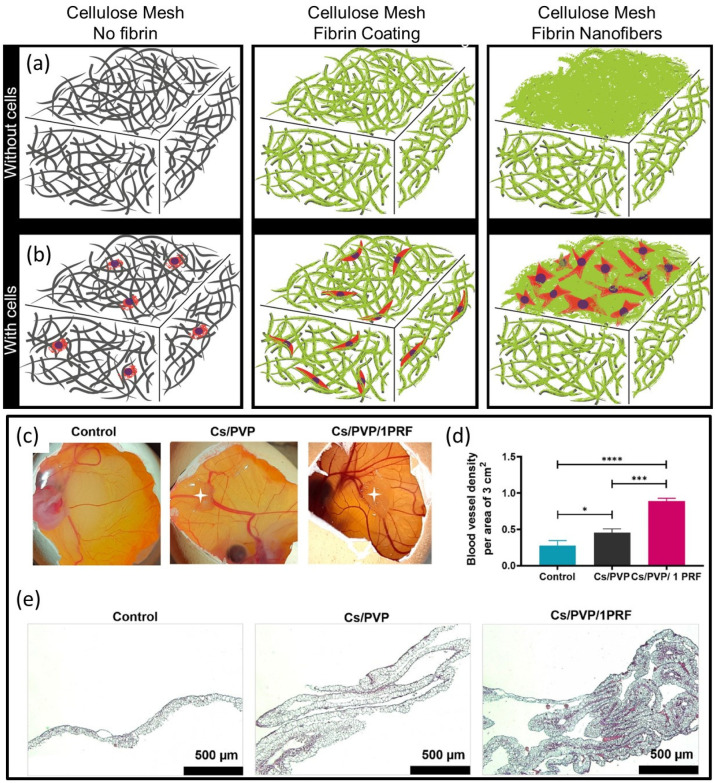
Schematic description of wound dressings fabricated from sodium carboxymethylcellulose combined with fibrin and seeded with dermal fibroblasts in vitro. (**a**) Series of porous structures of pure cellulose, cellulose coated with fibrin and cellulose coated with fibrin nanofibers with no cells. (**b**) Same as (**a**), but with cell seeding. (**c**) Images related to the angiogenic potential of various wound dressings assessed with the CAM assay. (**a**–**c**) Reprinted/adapted with permission from Ref. [48], 2018, MDPI. (**d**) CAM blood vessel density in different groups. (**e**) Histological images of CAMs associated with different studied groups stained with Masson’s trichrome (cell nuclei are red and collagen bundles are blue). Statistical significances are shown as * *p* < 0.05, *** *p* < 0.001 and **** *p* < 0.0001 in (**d**). Reprinted/adapted with permission from Ref. [48], 2022, Elsevier.

For muscular regeneration purposes, Wang et al. [44] designed cellulose nanocrystals, CNC-reinforced, fibrin nanocomposite hydrogels using unmodified CNCs and concentrated fibrinogen solutions. Fibrinogen adsorbed on CNCs such that CNCs decreased the viscosity of diluted fibrinogen solutions, and fibrinogen favored the alignment of CNCs under flow. Their CNC–fibrinogen mixtures that could gel easily exhibited strong potential as injectable formulations for the in situ formation of nanocomposite hydrogels favoring muscular tissue regeneration. Conventional bone repair therapies such as autologous and allogenic bone grafts have still several challenges to meet in bone reconstruction, along with complications to overcome. Tissue engineering targets new materials in regenerating damaged tissues rather than replacing them. Such materials, mostly in the form of composites, can act as templates for damaged tissues and function as an artificial extracellular matrix (ECM), expediting new tissue formation. Pathmanapan et al. [45] developed fibrin hydrogel incorporated with graphene oxide functionalized nanocomposite scaffolds for bone repair for enhanced bone tissue regeneration in vitro and in vivo. Their findings demonstrated that the nanocomposites enhanced levels of biocompatibility, alkaline phosphatase activity, and calcium deposits, signifying that they could be promising osteoinductive products for bone repair/regeneration. Dermal injuries and chronic wounds regularly restore themselves with scar formation. Treatment without scarring can be possible by pre-seeding a wound dressing with cells. Bacakova et al. [46] developed a wound dressing from sodium carboxymethyl cellulose, combined with fibrin and seeded with dermal fibroblasts in vitro. They tuned the chemistry and morphology of the wound dressings with the degree of substitution of hydroxyl groups. The wound dressing functionalized with two morphologically different fibrin structures enhanced the colonization of the material with human dermal fibroblasts. The fibrin nanofibers supported cell attachment and subsequent proliferation. The wound dressings functionalized with fibrin, especially in the form of a network of nanofibers, accelerated wound healing by supporting fibroblast adhesion and proliferation (see Figure 6a,b for schematic depictions of the materials).

Tigecycline is a broad-spectrum antibiotic that efficiently inhibits both Gram-positive and Gram-negative bacteria and can kill other antibiotic-resistant organisms. The controlled and sustained release of antibiotics is required for preventing mediastinitis (inflammation of the tissues in the mid-chest, or mediastinum). Sundaram et al. [47] developed tigecycline-loaded gelatin nanoparticles formed in situ and incorporated in a chitin–fibrin gel system designed to control bleeding and prevent bacterial infections at surgical sites. The authors evaluated the in vivo hemostatic potential of the nanocomposite gel in femoral artery and liver injuries in a rat model. The nanocomposite gels yielded the shortest clotting time and less blood loss in controlling blood ooze and pressured bleeding without compression, which is ideal for preventing mediastinitis after cardiac surgery. Tavakoli et al. [48] fabricated a freeze-dried chitosan (Cs)/polyvinylpyrrolidone (PVP) sponge containing platelet-rich fibrin as wound dressings. The dressings exerted effective antibacterial activity against *E. coli* and *S. aureus*. According to MTT and CAM assay studies conducted by the authors, the nanocomposite sponges significantly increased proliferation and angiogenic potential, respectively, with 97% in vivo wound closure after 14 days (see Figure 6c–e). The formation and growth of new blood vessels from existing blood vessels is known as angiogenesis, which is very important for the wound healing process, because novel blood vessels participate in the formation of granular tissue and carry nutrition and oxygen to the growing tissues. The CAM (Chick Chorioallantoic Membrane Assay) has a very dense capillary network; therefore, it is frequently used to investigate angiogenesis in vivo for testing wound healing materials. The results of the CAM assay presented in Figure 6c,d showed that angiogenesis around the Cs/PVP and Cs/PVP/platelet-rich fibrin-1PRF wound dressings had significantly increased compared with the control group. Finally, the sequence in Figure 6e shows the histological outcomes of CAM stained with Masson’s trichrome, which indicates the structure and thickness of CAM in different groups. As seen, the thickness of CAM for Cs/PVP and Cs/PVP/1PRF groups increased compared with the control group, which may be attributed to the increase in fibroblast proliferation and activity, which led to higher collagen deposition in relation to the control group.

Talukder et al. [49] prepared hybrid multilayered electrospun nanocomposites as wound dressing applications by electrospinning. The nanocomposite was a tri-layer membrane where the lower layer was made up of chitosan (CS)/polyvinyl alcohol (PVA) and fibrin (exhibiting properties of tissues and bleeding resistance regeneration), both of which were directly in contact with the burn skin wound, and a middle layer of PVA/sodium alginate (SA) (having antibacterial properties). The top layer consisted of gelatin with superhydrophilic properties.

**Figure 7:**
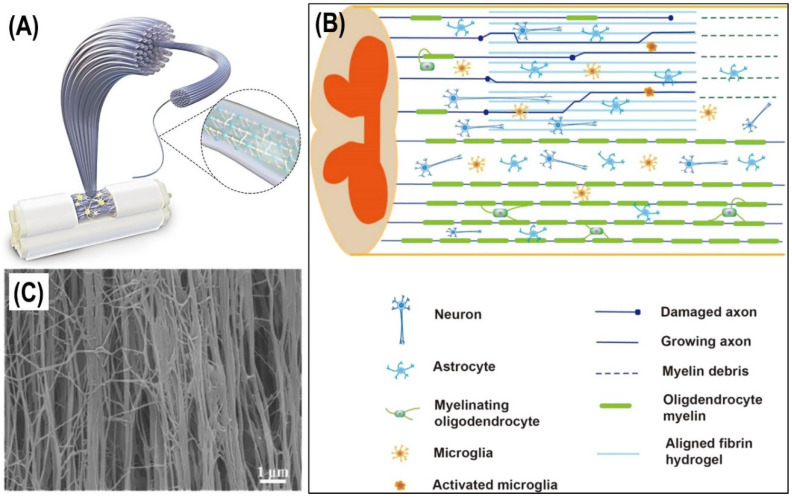
(**A**) Surgery process of canine traumatic hemisection T12 SCI model. (**A**) Diagram of AFG and (**B**) the SCI surgery and AFG transplantation process. Reprinted/adapted with permission from Ref. [53], 2022, Oxford University Press. (**B**) Schematic diagram of the axonal regrowth across the lesion site along the aligned nanofibers. Reproduced with permission from [52]. (**C**) Photomicrograph of electrospinning aligned fibrin hydrogel. Reprinted/adapted with permission from Ref. [54], 2020, Springer Nature.

Filová et al. [50] prepared nanofibers from a PVA/liposomes blend that was enriched with growth factors bFGF and insulin, and studied the release profile of the growth factors, and their effect on chondrocyte viability in vitro. The authors formed nanocomposites by embedding the nanofibers in a fibrin/type I collagen/fibrin composite hydrogel, and studied their impact on osteochondral regeneration in miniature pigs. Spinal cord injuries dislocate the long axonal tracts of the spinal cord and cause permanent neurological problems, for which effective fibrin-based therapeutic methods are still being developed [51]. Hierarchically aligned fibrin hydrogels were fabricated that could promote the neurogenic differentiation of stem cells in vitro towards peripheral nerve and spinal cord regeneration in rats [52]. In particular, aligned fibrin gels were used to repair a canine lumbar segment 2 hemisection spinal cord injury and to construct an aligned fiber bridge that supported cell adhesion in vitro, as depicted in Figure 7A,B. These nanocomposite gels in the form of aligned fibers (see Figure 7C) swiftly facilitated tissue invasion along the long axis of fibers in vivo, supporting the regrowth of axons in an oriented pattern connecting the rostral and caudal stumps. The directional axonal regrowth improved the recovery of motor functional behavior of spinal cord injury canines with aligned fibrin hydrogel impregnation.

Several new techniques have been used to increase the effectiveness of chitosan-based wound dressing materials to boost the regeneration of skin tissue at chronic burn wound sites. Kumar et al. [55] fabricated nanocomposite bandages of chitosan, gelatin, and fibrin, which performed better as compared with pure chitosan and some commercially available bandages. The nanocomposite bandages were macro-porous, biocompatible, and biodegradable, and absorbed excess exudate from the wound. The superior blood clotting and platelet activation achieved with the nanocomposites showed effectiveness toward wound dressing for blood-clotting disorders. The authors conducted in vivo burn wound repair studies with Sprague Dawley rats in which the enhanced deposition of collagen and re-epithelialization with the formation of an intact mature epidermis was seen.

## 5. Fibrin-Based Drug Delivery Systems

Fibrin-based drug delivery systems started to become popular in the early 1990s [56]. At that time, three potential delivery systems were identified, such as fibrin micro-particles with drug encapsulation by emulsification, reacting fibrinogen adsorbed on a suspended drug and thrombin in solution to form fibrin-coated drug systems and fibrin networks suitable as implants encapsulating drugs. It is imperative to briefly review what kind of proteins or growth factors (naturally occurring substances capable of stimulating cell proliferation, wound healing, and occasionally cellular differentiation) can interact with fibrin by nature. Epidermal cells and keratinocytes do not bind fibrin; growth factors bound within the fibrin clot include basic fibroblast growth factor (bFGF), VEGF165 transforming growth factor-b1, platelet-derived growth factor (PDGF-BB), IGF-1, and interleukin-1b. Other growth factors, such as bFGF, epidermal growth factor (EGF), PDGF (-A, -B), and VEGF165, are known to bind to heparin, which is bound within fibrin. Enzymes such as plasminogen, tissue plasminogen activator (tPA), plasminogen activator inhibitor, and thrombin also bind to fibrin [57].

**Figure 8:**
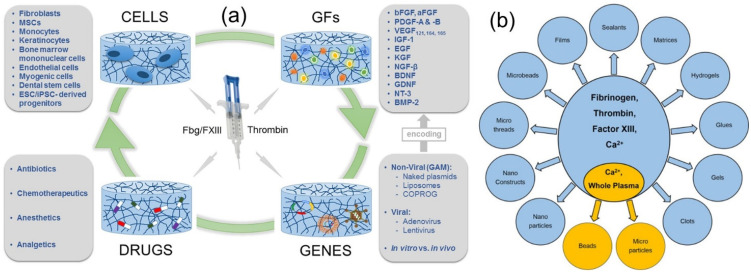
(**a**) Experimental and clinical delivery applications of fibrin in wound healing. Fibrin has been used to deliver cells, drugs, growth factors or gene vectors, and combinations thereof. GF: growth factor, Fbg: fibrinogen, FXIII: factor XIII, MSCs: mesenchymal stem cells, ESC: embryonic stem cell, iPSC: induced pluripotent stem cell, FGF: fibroblast growth factor, PDGF: platelet-derived growth factor, VEGF: vascular endothelial growth factor, IGF: insulin-like growth factor, EGF: epidermal growth factor, KGF: keratinocyte growth factor, NGF: nerve growth factor, BDNF: brain-derived neurotrophic factor, GDNF: glial-cell-line-derived growth factor, NT: neurotrophin, BMP: bone morphogenetic protein, GAM: gene-activated matrix, COPROG: copolymer protected gene vector. Reprinted/adapted with permission from Ref. [62], 2000, Elsevier. (**b**) Fibrin preparations useful in therapeutic delivery. Reprinted/adapted with permission from Ref. [60], 2001, Elsevier.

Fibrin scaffolds, known as “sealants,” are materials that mimic the final phases of blood coagulation, forming a stable, physiological fibrin clot that helps in wound repair. Extensive reviews on this can be consulted for further details on the surgical applications on fibrin-based scaffolds [58]. Fibrin sealants are shown to be biodegradable, and do not stimulate an inflammatory response, foreign body reaction, tissue necrosis, or fibrosis. Due to their structural and mechanical assets, as well as inherent biological importance, fibrin-based systems can be injected and polymerized in situ or can be functionalized by simply adding therapeutic molecules to fibrinogen–thrombin systems, or they can be designed as microspheres or nanoparticles to control the release kinetics of the delivered molecule. For instance, the efficacy of biologically active proteins in medical rehabilitation depends on specific delivery systems that should overcome severe problems related to wound healing proteins, such as short half-lives in body fluids and susceptibility to proteolysis and denaturation. Fibrin-based delivery systems were developed to encapsulate liposome proteins such as horseradish peroxidase (HRP) [59]. Liposomes protect proteins in aqueous environments; their encapsulation by fibrin ensures a depot system with sustained protein release. Figure 8a shows a schematic of the delivery of therapeutic biomolecules by fibrin matrices [60]. Cells, genes, drugs, and growth factors have all been embedded in fibrin matrices for sustained delivery, as schematically depicted in Figure 8a. On the other hand, the morphology of the fibrin matrix that can carry all these biological molecules and drugs is demonstrated in Figure 8b [59,61].

As a specific example, Park et al. [63] developed a fibrin- and gelatin-based drug delivery system to postoperatively improve the therapeutic effect of antibiotics in the ear cavity. They worked with four kinds of fibrin clot containing ampicillin, gentamicin, and ofloxacin, and the biologic assay was performed using *Bacillus subtilis*. Their fibrin–gelatin–gentamicin system featured antibiotic activity for up to 120 h against *B. subtilis*. Gentamicin was found to be released more slowly from the fibrin–gelatin matrix than the fibrin matrix alone. Their results showed that these fibrin-based release matrices can be clinically used in limited fields, such as the postoperative care of ear infection. Kumar et al. [64] prepared fibrin clots loaded with soluble tetracycline (TET) discs as a drug delivery matrix. As subcutaneous implants in mice, they degraded in 15 days. The authors observed a sustained release for up to 12 days into phosphate-buffered saline (PBS) and human serum. The fabricated fibrin discs were hemostatic and biodegradable in vivo, and in vitro release of a small molecule at a controlled rate allowed the authors to conclude that the materials could be good candidates as a drug delivery implant for short-term use. Sharma et al. [65] developed fibrin-based bio-printed constructs containing drug-releasing microspheres for neural tissue engineering applications. Similarly, Sharma et al. [66] fabricated fibrin-based bio-ink formulations combined with drug-releasing microspheres to be printed as tissues using human-induced pluripotent stem cell (hiPSC)-derived neural progenitor cells (NPCs). Microspheres ensured drug release in a sustained manner, delivering small molecules such as guggulsterone (a phytosteroid found in the resin of the guggul plant, Commiphora mukul). The printed tissues exhibited over 90% cellular viability 1 day post printing that then increased to 95%, 7 days post printing. The authors concluded that the printed tissues could promote the differentiation of neural tissue.

The local delivery of anticancer drugs is highly desirable, despite certain hurdles such as availability, biocompatibility, ease of operation, and efficacy. Fibrin gels (FBGs) have been suggested as attractive biomaterials for local treatment when loaded with different chemotherapeutics or with anticancer–drug polymer conjugates and nanoparticles. Such a controlled released system based on fibrin gels might counteract local recurrences or reduce the volume of inoperable tumors. Viale et al. [67] addressed this issue by studying the in vitro release of different formulations of doxorubicin from FBGs as a function of thrombin and Ca^2+^ ion concentrations. Doxorubicin was loaded in FBGs either alone or pre-incorporated in nanoparticles in which the fibrinogen and doxorubicin concentrations were found to have the greatest impact on controlled drug release. They did not, however, check their materials against tumor size reduction tests. A recent review on how autologous platelet-rich fibrin can be combined with other materials to ensure controlled drug and growth factor release can be referred to understand fibrin-based drug-releasing systems containing growth factors and peptides that provide tissue regeneration [68]. Willerth et al. [69] explored a number of peptide sequences with varying affinity for nerve growth factor (NGF) and employed them as affinity-based drug delivery systems from fibrin matrices. The authors experimented with the fibrin matrices containing these peptides and NGF to deliver to biologically active NGF using a chick dorsal root ganglia model. They also developed a mathematical model to better understand how to tailor the affinity of a drug delivery system for a target protein drug. In another study [70], glutaraldehyde-treated dexamethasone-containing cylindrical fibrin gels fabricated by the thrombin-induced polymerization of fibrinogen in the presence of the drug induced cross-linking of the gels and modification of the pore structure. The release of dexamethasone was studied by measuring the diffusion coefficient of the drug over the treated and untreated gels. Biodegradation experiments indicated that the glutaraldehyde-treated gels were resistant to digestion by plasmin, but untreated gels were digested, and the digestion rate was accelerated by plasmin. Nanostructured fibrin systems were made by water-in-oil emulsion templating without the use of any surfactants to form fibrin nanotubes (FNTs) and fibrin nanoparticles (FNPs). Both were found to be enzymatically degradable, with no long-term toxicity effects [71]. The authors used them to test the delivery of tacrolimus, an immunosuppressive drug which is widely used to prevent the initial phase of tissue rejection during allogenic transplantation surgeries. The fibrin nanomaterials achieved drug encapsulation efficiency of 66% with the in vitro release in PBS for over a period of one week at the physiological pH of 7.4. The authors reported that under an acidic pH, the drug release was very slow, rendering them appealing for oral–intestinal drug administration. The in vivo drug absorption tests on Sprague Dawley rats showed the sustained release of tacrolimus via both oral and parenteral delivery routes. As a suggestion for future studies, the authors have proposed exploring the delivery of specific growth factors in tissue engineering scaffolds.

## 6. Fibrin and Cell Delivery

**Figure 9:**
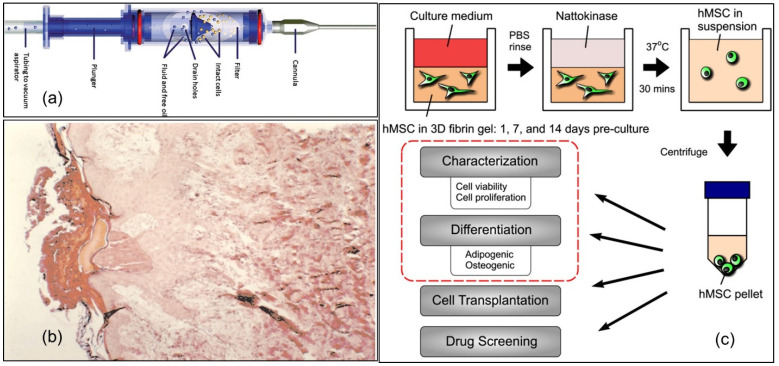
(**a**) Schematic of a modified syringe-based device. The device was designed to harvest small volumes of autologous adipose tissue for cosmetic surgical applications. It aspirates fat at low vacuum into a sterile chamber. Once inside, the fat cells are concentrated by filtration. Fluids and free oil are drawn into a waste canister. Reprinted/adapted with permission from Ref. [73], 2013, Elsevier. (**b**) Biopsy taken 6 days following the application of fibrin glue suspended keratinocytes combined with allogeneic overgrafting. Histology demonstrates the initial integration of allogeneic dermal elements in the reconstituted neoskin. Reprinted/adapted with permission from Ref. [75], 2014, Mary Ann Liebert, Inc. (**c**) Illustration depicting the methods by which MSCs were extracted from 3D fibrin gels. Reprinted/adapted with permission from Ref. [76], 2004, Springer Nature.

In this section, we discuss the use of fibrin for the delivery of cells and focus on the various strategies that have been proposed to tune the release rate and to enhance the regenerative process during wound healing. We also explore other recent advances such as the dual delivery of cells and growth factors (GFs) [72]. As we have conferred throughout this review, fibrin participates in the earliest phase of wound healing, whereas the proliferative phase is controlled by cells that mediate granulation and angiogenesis. Zimmerlin et al. [73] developed bone-marrow-derived multipotent mesenchymal stromal cells (MSCs) suspended in fibrin glue and used as an aerosol (see Figure 9a) for patients with acute wounds related to skin cancer and non-healing lower extremity wounds. SVF cells remained viable after application and proliferated for up to 3 weeks, when they reached confluence and adipogenic differentiation. Under angiogenic conditions, in which new blood vessels form from pre-existing vessels, SVF cells formed endothelial vWF+ (von Willebrand factor, a blood glycoprotein involved in hemostasis), CD31+ (traditionally a marker for endothelial cells,) and CD34+ (transmembrane phosphoglycoprotein protein encoded by the CD34 gene in humans, mice, rats, and other species) tubules surrounded by CD146+ (human umbilical cord perivascular cells) and α-smooth muscle actin+ perivascular/stromal cells. As a result, healthy tissue growth occurred, which is good for wound healing, without in vitro expansion. Similarly, Zhang et al. [74] investigated the delivery of bone-marrow-derived mononuclear cells (BMNCs) by an injectable PEGylated fibrin system that covalently binds hepatocyte growth factor (HGF) that was expected to enhance the rate of cell engraftment and improve cardiac function. Using several mice models, the authors showed that the cell prevalence rate at 4 weeks increased 15-fold in hearts receiving the fibrin matrix + HGF + cell delivery (*p* < 0.01), which was accompanied by the lowest levels of apoptosis and the highest LV (left ventricular) function recovery among the treated groups.

Adherence of transplanted cells to the wound bed particularly in the presence of potential wound contamination is very important for wound healing. In acute burn wounds, as well as in chronic wounds, fibrin-matrix-embedded cultured human autologous keratinocytes were shown to adhere to wound beds and spread over the wound, resulting in the re-epithelialization of both acute and chronic wounds (see Figure 9b) [76]. Carrion et al. demonstrated a safe and efficient method to retrieve mesenchymal stem cells from three-dimensional fibrin gels [75]. Mesenchymal stem cells (MSCs) exhibit multipotent features that make them ideal for potential therapeutic applications. Three-dimensional (3D) culture systems that more closely resemble the physiological environment of MSCs and other cell types are generally employed to support and maintain the cell phenotypes, as illustrated in Figure 9c. For this purpose, fibrin has been utilized as the provisional extracellular matrix for wound healing. Although fibrin enables cells to adhere, spread, proliferate, and undergo complex morphogenetic programs, new processes are needed to safely retrieve cells encapsulated within fibrin hydrogels to perform additional analyses or use the cells for wound healing. The authors used nattokinase extraction—nattokinase is a serine protease of the subtilisin family that has a strong fibrinolytic activity—and the extracted cells were viable but also retained their proliferative and multilineage potentials. The authors discussed the potential application of the cells for in vivo implantation into wounds, but did not demonstrate a wound healing model.

Autologous fibrin scaffolds (AFSs) encapsulating both cells and specific growth factors can be used as a promising biocompatible scaffold for tissue engineering. De La Puente et al. [77] developed a biocompatible and implantable AFS, using autologous porcine plasma without fibrinogen concentration methods, as an alternative to commercial fibrin [78]. Developed AFSs were loaded with fibroblast growth factor microspheres (with an optimal release period from a clinical point of view) and dermal fibroblasts that proved to have good cellular distribution, viability, adhesion, and proliferation. However, they did not demonstrate a wound healing application for the dual cell/growth-factor-loaded fibrin matrices. In a recent study, Singaravelu et al. [79] fabricated medicated wound dressing material with highly interconnected pores, mimicking the function of the extracellular-matrix-containing cells. They utilized keratin (K), fibrin (F), and gelatin (G) to create a composite scaffold (KFG-SPG) by employing a freeze-drying technique and incorporated the mupirocin (D) drug as well as the NIH 3T3 fibroblast and human keratinocytes (HaCaT) cells. Unfortunately, they did not demonstrate any wound model tests.

An important medical problem following spinal cord injury (SCI) is the poor cell survival and uninhibited differentiation following transplantation. Johnson et al. [80,81] studied the feasibility of enhancing embryonic-stem-cell-derived neural progenitor cell (ESNPC) viability. To do so, they induced cell differentiation towards neurons and oligodendrocytes by embedding the ESNPCs in fibrin scaffolds containing growth factors (GF) and a heparin-binding delivery system (HBDS) in a subacute rat model of SCI. They showed that a combination of the NT-3, PDGF, and fibrin scaffold (with or without HBDS) enhanced the total number of ESNPCs within the spinal cord lesion 2 weeks after injury. Moreover, the inclusion of the HBDS with a growth factor caused an increase in the number of ESNPC-derived NeuN-positive neurons, indicating the ability of fibrin scaffolds and the controlled release of growth factors to boost the survival and differentiation of neural progenitor cells following transplantation into an SCI model. Human neural stem/progenitor cells (hNSPCs) are important for treating central nervous system (CNS) trauma by secreting trophic factors and differentiating into mature CNS cells; however, many cells die after transplantation. This problem may be circumvented by the inclusion of a fibrin-based scaffold. Arulmoli et al. [82] found that fibrin generated from salmon fibrinogen and thrombin stimulated greater hNSPC proliferation than mammalian fibrin. Fibrin scaffolds degraded within a few days in vivo, retaining the beneficial properties of fibrin, but then degraded more slowly to provide longer support for hNSPCs. Furthermore, composite scaffolds of salmon fibrin, along with hyaluronic acid (HA) with and without laminin, were polymerized more effectively than fibrin alone, transforming into hydrogels exhibiting physical properties very close to the brain tissue. Novel scaffolds impregnated with cord-blood-derived endothelial cells enabled vascularization and inhibited the loss of hNSPCs. Lei et al. [83] studied the fibrin-mediated gene transfer process by embedding pDNA in a fibrin hydrogel during fibrin polymerization. They used two modes of gene transfection, with cells immobilized on both the surface and inside the hydrogel. They reported that upon embedding the cells within the fibrin matrix, lipofectamine-induced cell death decreased significantly, especially at low-target cell density. They also observed that for efficient gene transfer in a dose-dependent manner, fibrin degradation should take place. Based on their experimental work, fibrin could represent an ideal matrix to deliver genes in an efficient, cell-controlled, and spatially localized fashion [83]. It has been argued that the outcome of fibrin cell delivery can, in part, be attributed to the relative concentrations of fibrinogen and thrombin solutions (i.e., formulations) and the structure of the final 3D fibrin clot [84]. The proliferation of human mesenchymal stem cells (hMSCs) within 3D fibrin clots in vitro shows such formulation dependent variation. For instance, the fibrinogen solution, not thrombin, was found to have a more central role on hMSC proliferation, with dilute fibrinogen solutions promoting better hMSC proliferation, yielding more open, homogeneous microstructures. It can be concluded that the concentrations of fibrinogen and thrombin solutions must be adjusted carefully, depending on the wound treatment by cell delivery, because this affects 3D fibrin clot structure and cell proliferation.

## 7. Fibrin and Skin Reconstruction

Probably the most commonly utilized and studied biodegradable synthetic polymers for skin closure and suturing are cyanoacrylate monomers (CAs). The hemostatic activity of CAs has been reported in many studies [85,86,87,88,89]. Unlike CAs, fibrin-based skin closure materials are more effective in controlling the leakage of air, blood, and fluid during a wide variety of thoracic and cardiovascular procedures, and might be beneficial to other surgeons with a better tissue compatibility, biodegradability, and efficacy when applied to wet surfaces [90,91]. The ultimate objective of skin tissue regeneration is to develop artificial skin replacements for the restoration of damaged or missing skin in patients, as well as to enhance wound healing processes. To this effect, fibrin-based materials should be explored and developed further for skin construction strategies. This is driven by fibrin’s remarkable skin repair capacity over CAs, such as intrinsic healing properties, adaptable to biomaterial designs from its fibrinogen and thrombin precursors, and tunable physico-chemical features. Notably, despite the breadth and depth of recent research on fibrin(ogen), many outstanding issues remain. For example, molecular mechanisms of lateral aggregation and branching in fibrin polymerization have not been completely and mechanistically identified. The majority of research on fibrinogen and fibrin has been on mice models; thus, much remains unknown about their roles in other mammals. Many of the binding associates of fibrin(ogen) have been identified, but their functions are still not very clearly identified, as indicated by Weisel and Litvinov [92]. Fibrin’s poor mechanical properties can be efficiently improved by combining it with other biomedical biopolymers, such as polyethylene glycols [93]. For instance, Sánchez-Muñoz et al. [94] demonstrated that endothelial cells (CD31 and von-Willebrand-factor-positive) proliferated and organized themselves into capillary-like structures within the fibrin-based avascular-engineered skin equivalents. They constructed a new model of endothelialized fibrin-based skin substitutes that exhibited complete epithelization by squamous cells (AE1/AE3 cytokeratin-positive) with intracytoplasmic keratohyalin granules, hyperkeratosis, and parakeratosis. They concluded that their material was a useful tool for regenerative medicine.

**Figure 10:**
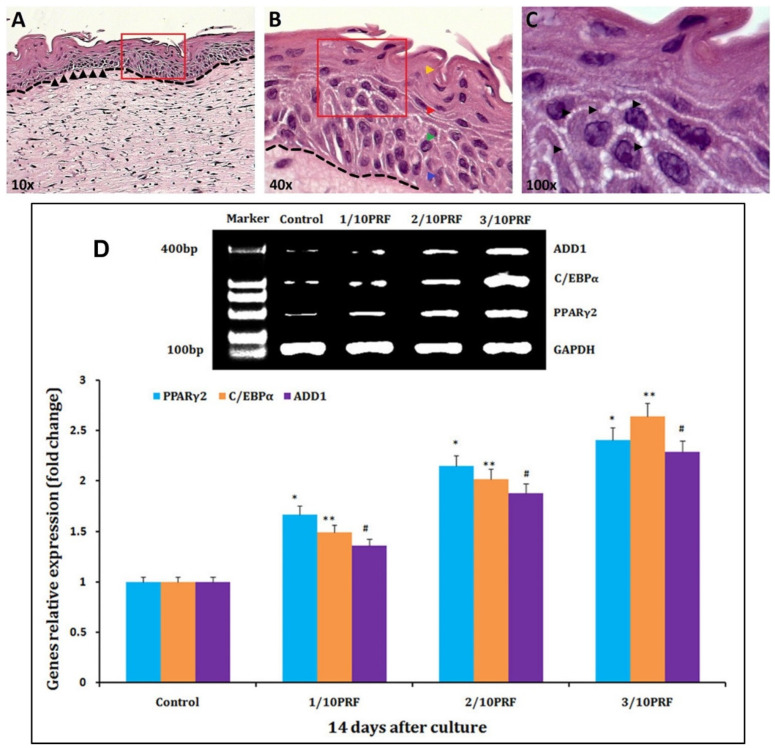
Histological features of skin organotypic cultures (ORGs) determined by H&E, after 21 days. (**A**) Whole structure of the ORGs (10×). A clear dermal–epidermal separation (dashed line) and a possible basal membrane (black head arrows) can be seen. (**B**) The enhanced area (40×) indicated by the red inset in (**A**). Four differentiation stages of the epidermis: the basal layer (blue head arrow), the spinous layer (green head arrow), the granular layer (red head arrow), and the horny layer (yellow head arrow), and the morphological associated changes in keratinocytes through the different layers of the epidermis. (**C**) Enhanced (100×) image indicated by the red inset in (**B**). The black arrows indicate hemidesmosome-like structures between keratinocytes. (**A**–**C**) Reprinted/adapted with permission from Ref. [97], 2016, Elsevier. (**D**) mRNA levels of PPARγ2, C/EBPα, and ADD1 mRNA, which are adipogenic marker genes, are higher in the platelet-rich fibrin (PRF) groups than in the control group after 14 days of culture. * *p* < 0.01, ** *p* < 0.01, # *p* < 0.01. Reprinted/adapted with permission from Ref. [98], 2017, Impact Journals, LLC.

Novel fibrin-based materials for skin constructs also compete with collagen-based materials. In fact, both collagen and fibrin are widely used in tissue engineering due to their function towards in vivo tissue formation. Comparative studies have been conducted on both materials using the growth of rat dermal fibroblasts or dermal fibroblasts and epidermal keratinocytes together in collagen and fibrin constructs, respectively, with and without the reinforcement of electrospun poly(lactic acid) nanofiber mesh [95]. Cell proliferation, gel contraction, and elastic modulus of the constructs were measured on the same gels at multiple time points during a 22-day culturing period using multiple non-destructive techniques. Keratinocytes and fibroblasts demonstrated different cellular activities within the fibrin- and collagen-based gel–skin constructs. Collagen gels with keratinocytes and fibroblasts demonstrated reduced mechanical strength. Fibrin gels with keratinocytes and fibroblasts exhibited much better and more stable mechanical strength. The use of nanofiber mesh to support the gels increased fibroblast proliferation when keratinocytes were absent [95,96]. Morales et al. [97] used an in-house human dermal-epidermal model to construct human fibrin-based dermal–epidermal organotypic skin cultures (ORGs) exhibiting similar histological characteristics to native skin and articulating specific differentiation epithelial proteins. More specifically, they used human primary keratinocytes and fibroblasts from a typical body region. All ORGs were built with high concentrations of calcium (1.16 mM) throughout the culture tests. ORGs were used to perform corrosion and irritation tests under OECD Test Guides (OECD TG 431 and 439; see Figure 10). In a unique study, nanofat-derived stem cells with platelet-rich fibrin were shown to improve facial contour remodeling and skin rejuvenation after autologous structural fat transplantation [96]. Nanofat-derived stem cells (NFSCs) were isolated, mechanically emulsified, cultured, and within platelet-rich fibrin (PRF), the proliferation and adipogenic differentiation of NFSCs were enhanced in vitro. Based on 77 test group patients including controls, who underwent traditional autologous fat transplantation, facial soft tissue depression symptoms and skin texture were improved to a greater extent after fibrin-mediated nanofat transplants than after traditional transplants. The results indicated that fibrin-mediated nanofat transplants could represent a safe, highly effective, and long-lasting method for remodeling facial contours and rejuvenating the skin [98].

Encouraging results on wound healing enhancement using fibrin–fibronectin matrices have been reported in recent studies [99,100,101]. Plasma fibrinogen (F1) and fibronectin (pFN) polymerize to form the fibrin; about 90% of plasma F1 has a homodimer pair of γ chains (γγF1), and 10% has a heterodimeric pair of γ and more acidic γ′ chains (γγ′F1). Jara et al. [97] synthesized a new fibrin matrix based on a 1:1 (molar ratio) complex of γγ′F1 and pFN in the presence of thrombin and recombinant factor XIII (rFXIIIa), in which the fibrin nanofibers were decorated with pFN nanoclusters. The authors labeled this new system as γγ′F1:pFN fibrin, which enhanced the adhesion of primary human umbilical vein endothelium cells (HUVECs) and primary human fibroblasts. Particularly, HUVECs in the new fibrin matrix exhibited a starkly enhanced vascular morphogenesis compared with a standard fibrin-based matrix that demonstrated an apoptotic growth profile. Furthermore, mouse dermal wounds sealed by γγ′F1:pFN fibrin exhibited accelerated and enhanced healing. As can be seen in the abovementioned examples, bioengineering human skin can allow the efficient treatment of patients with severe skin defects. However, more work will be needed to optimize many different properties of these skin substitutes, including the optical and biomechanical properties of these fibrin-based constructs. In a particular system, researchers have developed fibrin–agarose biomaterials (acellular, dermal skin substitutes, and complete dermoepidermal skin substitutes) and studied their optical and biomechanical properties using the inverse adding–doubling method and tensile tests, respectively. Their analysis showed that the optical properties of the fibrin-based materials resembled the optical behavior of the native human skin in terms of absorption and scattering properties. The properties of the fibrin-based dermoepidermal substitutes were the same even after 7 to 14 days in culture compared with native human skin. Similarly, the biomechanical parameters did not differ from the control skin for traction deformation, stress, and strain at fracture break [100,101,102,103]. Table 1 demonstrates that there are many studies on skin reconstruction based on new-generation fibrin-based materials. A large proportion of these studies did not report or study the biomechanical properties of these skin substitutes, even though this aspect is exceedingly important for compatibility with skin texture, permeation and swelling, and conformity to the various natural skin stress–strain levels.

Table 1 clearly indicates that fibrin-based material systems are among the newest autologous clinical treatments that can accelerate the wound healing process, particularly wound epithelialization. Although different animal models have been usefully implemented, many of these developments still need to be tested under clinical conditions. Moreover, half of the studies reviewed in Table 1 did not use any cells or growth factors integrated into the fibrin-based skin repair materials. Inflammation reactions such as hyperemia, pain, hyperthermia, and edema in the wound sites were reduced [120,121]. Another unique application of fibrin-based materials is the soft tissue closure. It was demonstrated that, for instance, in meningomyelocele repair, which is the most common and complex birth defect of the central nervous system, consecutive separate closures of the neural placode, dura mater, lumbar fascia, subcutaneous layer, and skin need to be conducted. The use of a fibrin-enriched bovine pericardial patch at the fascial level—between the dural sac and the skin—provided sufficient soft tissue coverage in meningomyelocele repair surgery [122].

## 8. Applications of Drug-Loaded Fibrin-Based Materials

Bioactive complexes, including growth factors, cytokines, drugs, and nucleic acids, can be easily encapsulated in fibrin-based matrices by mixing them with fibrinogen or thrombin. Generally, fibrin matrices have a porous nature, regardless of the thrombin and fibrinogen ratios. It has been reported that increasing fibrinogen and/or thrombin concentrations densifies the structure of fibrin-based materials and reduces their degradation rate, thus slowing down the diffusion of loaded drugs [123]. Although the widespread use of fibrin-based materials in biotechnology and the clinic has been increasing rapidly, more research and development efforts are still needed in areas such as the better control over mechanical properties (tailoring elastic modulus–elongation properties), slowing down degradation, or retaining a more sustained release capability [124]. Fibrin can not only bind, retain, and release a diverse scale of biomolecules and drugs, but also induces the right environment (i.e., porous, fibrous, and larger effective surface area) within other biodegradable/natural polymers, which can have profound effects on cell behavior and wound repair [124,125]. It is with this motivation that this section presents, reviews, and discusses a number of recent published studies on drug-loaded fibrin-based systems and associated release characteristics designed for wound healing and repair [126]. Notable studies have been compiled in Table 2.

As can be seen from the comparative table summary above (Table 2), fibrin-based materials such as gels, films, or nanoparticles have been extensively investigated as media for local and prolonged drug delivery. The effective local delivery of antibacterial substances is important preoperatively in patients with implanted medical devices, or postoperatively for deep wounds. It is known that the prolonged local application of antibiotics is often not possible or simply insufficient. Biofilm formation and antibiotic resistance are also major hindrances in antibacterial therapy. These issues were addressed by Rubalskii et al. [146], who tested the biocompatibility of bacteriophages embedded in a fibrin glue, recorded the release of bacteriophages from fibrin scaffolds, and measured their antibacterial activity. Efficient PA5 bacteriophages were released from the fibrin glue during 11 days of incubation in a liquid medium. Released PA5 bacteriophages efficiently killed *Pseudomonas aeruginosa* PA01. The sustained release of soluble growth factors (GFs) in hydrogel tissue constructs stimulates the migration and proliferation of embedded cells which can act as scaffolds for neural tissue regeneration [147]. For instance, Lee et al. [145] prepared fibrin gels containing vascular endothelial growth factor (VEGF) as an artificial neural tissue. They also employed the bio printing of these materials embedded with cells. They did not report any release kinetics of VEGF, but concluded that up to 3 days, VEGF release took place with cell proliferation. Similarly, Sacchi et al. [146] developed tunable fibrin-based scaffolds to accurately control the dose and duration of VEGF protein delivery in tissues. They optimized the sustained delivery of fibrin-bound VEGF to ensure healthy, stable, and efficient angiogenesis and improved perfusion of ischemic tissues, without genetic modification. Again, they did not demonstrate release kinetics, but indicated that VEGF release lasted for up to 4 weeks [148].

Losi et al. [149] designed poly(ether)urethane–polydimethylsiloxane–fibrin composite scaffolds for the controlled delivery of pro-angiogenic growth factors and tested the tissue response. They also investigated VEGF protein delivery from these composite scaffolds. They characterized cumulative release profiles for up to 14 days and proposed their materials for the treatment of ischemic tissue and wound healing. In order to test the clinical efficacy of the prolonged delivery of TG-PDGF.AB (a platelet-derived growth factor with transglutaminase cross-linking) released locally from a sprayed fibrin matrix that was developed for an ischemia model, Mittermayr et al. [150] studied the impact of this system on tissue necrosis and the potential to induce functional angiogenesis. Release rates up to 80 h have been demonstrated; the fibrin matrix conjugating the PDGF.AB enabled the controlled local release of the growth factor and enhanced ischemia-induced tissue loss. Fibrin sealants have been proposed as depot matrices for substances due to their biocompatibility, advantageous biological properties, and widespread use in wound healing. The study conducted by Morton et al. [151] utilized fibrin sealants as sustained release matrices for thrombin, fibronectin, and DNA. All medicinal proteins were allowed to be released for a continuous period of 10 days. The controlled release of neurotrophin-3 from fibrin gels for spinal cord injury was studied by Taylor et al. [152]. They modified the fibrin gels with heparin to bind the medicine for sustained release; as a result, the gels stimulated neural outgrowth from chick dorsal root ganglia by up to 54% compared with original fibrin. In addition, upon monitoring the release of neurotrophin-3 for 9 days continuously, they showed that the medicated fibrin gels increased neural fiber density in spinal cord lesions compared with unmodified gels [152]. Finally, it has been acknowledged that the sustained dual drug delivery of anti-inhibitory molecules for the treatment of spinal cord injury can lead to chondroitin sulfate proteoglycans (CSPG)-associated inhibition and allow for improved axon growth [153]. Indeed, the sustained delivery of both chondroitinase ABC and Nogo-A extracellular peptide residues 1–40 (NEP1–40) over one week in vitro by fibrin scaffolds implanted into injured rat spinal cords has proven very useful by reducing the development of glial scars and by increasing the number of axons near the injury site [153].

## 9. On the Mechanical Properties of Fibrin Fibers

As hypothesized, when blood clots form as wound healing initiates, the clot itself should sustain wound healing; hence, it has to have specific mechanical properties such as being remarkably extensible and elastic. This is achieved with fibrin fibers, and the mechanical properties of fibrin fibers depend on the mechanical properties of the individual fibrin monomers. The fibrin monomer is defined by three key structural attributes: the coiled-coil connectors, the folded globular nodules, and the relatively unstructured αC regions [19,154]. Fibrin is a viscoelastic protein exhibiting both elastic and viscous properties. The elasticity (or stiffness) is described by reversible mechanical deformation, and the viscosity (or plasticity) is defined by irreversible force-induced deformation. Fibrin-laden clots have an elastic component one order of magnitude higher than the viscous component, even though the viscous component grows rapidly at higher deformation rates. Curiously, various creep experiments have shown that even after continued changes, with strain measured over time after the application of stress, some clots do not change in stiffness, which should indicate that fibrin is a self-healing fiber network, most likely because the knob–hole bonds holding the structure together are reversible [17]. Further rheometry-based quantification of fibrin viscoelasticity is presented in [26,27]. Table 3 compares the elasticity of some protein-based fiber network structures [8,17].

As shown in Table 3, cross-linked fibrin fiber has both a higher elastic modulus and elongation at break value. It has been proposed that the α-C region plays a significant role in the inter- and intra-linking of fibrin molecules and protofibrils, providing fibrin fibers with increased stiffness and elasticity [155]. Other natural fibers also feature high elasticity and elongation values, such as the catching thread of spider webs, fibronectin fibers connecting cells, or elastin fibers in the extracellular matrix. Large elongation at break values mean high toughness (the area under a stress–strain curve), which is measure of how much energy a fiber can absorb before failing. Such fibers can absorb sudden, large forces or bursts of kinetic energy without breaking, such as the pulling of fibers when a fly is entangled in a spider web. In clots, as fibrin fibers form, energetic blood flow is maintained and the tough fibers can absorb these flow forces and the energy without breaking.

Recent studies on the mechanical response of non-cross-linked fibrin fibers indicated a nonlinear mechanical response of networks that continually changes in response to repeated large-strain loading [156]. It was argued that such a dynamic mechanical response originated from a shift in the characteristic nonlinear stress–strain relationship to higher strains. This means that the imposed loading delays the occurrence of strain stiffening instead of weakening the fiber network. This was attributed to the tenacious lengthening of individual fibers caused by an interplay between fiber stretching and fiber buckling when the fibrin fibers were repeatedly strained. Inversely, the covalent cross-linking of fibrin fibers inhibited the shift in the nonlinear response, signifying that the molecular origin of fiber lengthening could be the slipping of monomers within the fiber network. Amazingly, this demonstrates that fibrin fibers exhibit unique internal plasticity, whose mechanical response can easily adapt to external loading conditions [156]. In fact, very recent studies based on atomistic simulations and high-resolution microscopy suggest that the unique mechanical behavior of fibrin fibers and clots could be due to a new protofibril element made up of a nonlinear spring network, resulting in the force extension behavior of fibrin fibers [157]. Studies have further shown that complex interactions exist among protofibrils, including Coulombic attraction and other binding forces. Such models are important to predict the behavior of fibrin fibers as well as fibrin clots at small strains, large strains, and close to the break strain. As a result, new adaptive wound healing materials can be designed. Using the standard tensile testing of non-cross-linked fibrin polymers in vitro and in silico, researchers have obtained stress–strain curves [156] for fibrin fibers characterized by elastic deformations with a weaker elastic response for strain rates lower than 160%. This was attributed to the unraveling of αC tethers and straightening of fibrin protofibrils. The fibrin fibers demonstrated a stronger response for strain rates higher than 160% due to unfolding of the coiled-coils and γ nodules in fibrin monomers. Fiber rupture was also measured beyond strain rates of 210%; the mechanism was attributed to dissociation of the knob–hole bonds and the rupture of D:D interfaces. Due to such measurements and supporting modeling studies, fiber elongation was confirmed to be associated with protofibril dehydration and the sliding mechanism to create an ordered protofibril array [158].

Using blood collected from voluntary donors, Kim et al. [159] studied the mechanical properties of fibrin networks, with and without cells, formed under wild-type and hemophilic conditions. The three-dimensional morphology of each fibrin network was reconstructed from confocal microscopy image sections, and the images were used to formulate microstructure-based models to analyze the relationship between the structure and mechanical properties of the fibrin networks. The mechanical properties were evaluated by examining the fiber network responses to uniaxial tensile and shear stresses. This was performed to simulate the impact of blood flow on the fibrin network. Both the model and experiments confirmed fiber network alignment under load, and the results were in agreement with other studies. It was shown that a nonlinear worm-like chain model correctly predicted both the elastic properties of the networks and the alignment of the fibers as the clot sample was stretched. In a recent study, Li et al. [160] investigated the influence of fibrinogen glycation and the fibrin fiber diameter on the mechanical properties of single fibrin fibers using combined atomic force microscopy/fluorescence microscopy.

**Figure 11:**
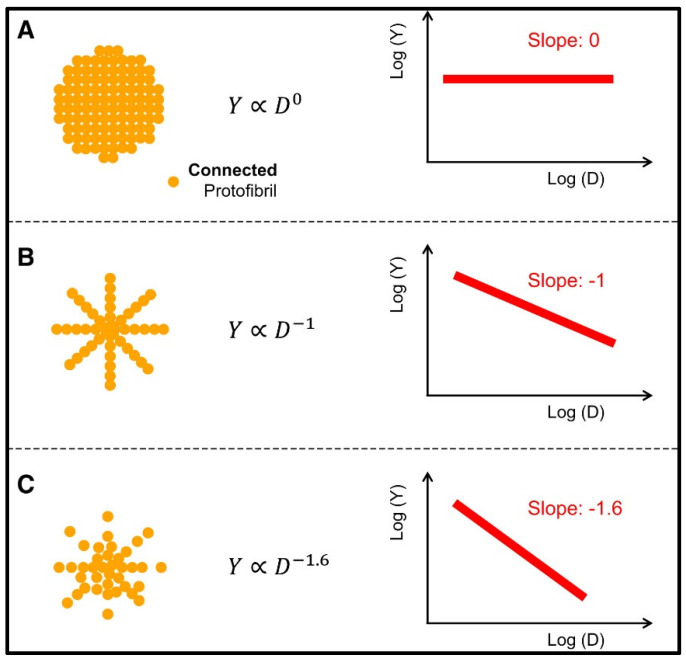
Cross-section fibrin fiber models and their corresponding stretch modulus. (**A**) A fiber with uniformly connected protofibrils has a stretch modulus that is independent of diameter, D. (**B**) A fiber with a bicycle-spokes-like cross-section can have a stretch modulus that decreases as D^−1^. (**C**) Based on experimental observations, however, the stretch modulus scales as D^−1.6^. This may indicate that the density of connected protofibrils will decrease with increasing fibrin diameter, D, as D^−1.6^. Reprinted/adapted with permission from Ref. [160], 2021, Elsevier.

They used individual fibrin fibers formed from blood plasma obtained from uncontrolled diabetic patients as well as age-, gender-, and body-mass-index-matched healthy individuals. They found that fibrinogen glycation had no notable methodical effect on the single-fiber modulus, extensibility, or stress relaxation times. Conversely, they reported that the fiber modulus decreased significantly with the increasing fiber diameter. Thin fibers were found to be 100 times stiffer than thick fibers, which contradicts the fact that the modulus is a material constant and does not depend on the sample dimensions (diameter) for single or homogeneous materials. They concluded that fibrin fibers do not have a homogeneous cross-section of uniformly connected protofibrils, but the density of protofibril connections probably decreases with increasing diameter, making thin fibers denser and with more strongly connected protofibrils than thick fibers. As such, it might be possible that clots consisting of fewer thick fibers will be more easily dissolved than those that consist of many thin fibers, which is consistent with experimental and clinical observations. By definition, a homogeneous fibrin fiber structure should feature a uniform density of equally connected protofibrils in the radial direction (as shown in Figure 11A). As discussed above, if the fibers do not have a homogeneous cross-sectional composition, it can be postulated that the density of the protofibrils and/or the protofibril connections within a fiber vary with diameter (as shown in Figure 11B,C). In addition, reports in the literature detail new test methods in which the mechanical behavior of macroscopic clot thrombus material exposed to compression or tension force was investigated [160]. These methods were developed to measure the mechanical properties of bulk clot material and subsequently further understanding of the influence of polymerization conditions on the mechanical properties of clots. Additionally, these new test methods enabled the measurement of not only the elastic properties of clots, but also the tension and strain at rupture of a thrombus bulk material. The measurements showed increased strength in aged blood material that was dominated through the fibrin architecture. From the results of the compression tests, it was concluded that the addition of barium sulphate leads to an increase in thrombi strength. Furthermore, the elastic moduli of the bulk thrombi differed significantly from those of single fibers [161].

As discussed in previous reports, the characterizations of not only pure fibrin fiber mechanical properties, but also biomedical materials that contain fibrin fibers such as films, gels, or hydrogels and mucoadhesives, are equally important for the longevity of wound treatment and drug release [38,91,162,163,164,165]. The importance of mechanical properties of materials for chronic wound care has recently been acknowledged as vital for the biochemical function of these materials [166]. Although the viscoelasticity and softness of wound dressing materials are hypothetically essential for creating adaptive cellular niches, the underlying mechanically regulated wound healing mechanism remains intangible. It has been demonstrated that softer and highly viscoelastic hydrogels can facilitate cell proliferation, granulation formation, collagen aggregation, and chondrogenic ECM (extracellular matrix) deposition [167]. Moreover, the use of fibrin fiber in scaffolds that are suitable for cartilage tissue engineering applications requires more attention to mechanical properties of these materials, and fundamental understanding of fibrin-based soft composites’ mechanical properties and the underlying molecular mechanisms is vital for defining whether these biomaterials are potentially suitable for wound repair applications [168,169,170,171,172,173].

## 10. Conclusions

It is beyond any doubt that fibrin fibers facilitate and enhance the process of wound healing. They particularly regulate wound closure, the rate of healing, and aid in strengthening the wound site. Fibrin fibers, as well as fibrin-based soft biomedical composites, contribute to the wound’s ability to resist infection and other wound complications; influence the extent of inflammation at the wound site; and, in the case of skin wounds, affect the appearance of the healed wound site. The arguments presented here are the reasons why many commercial fibrin sealants have been developed and are extensively used today in surgical applications. Fibrin-based materials proved useful in a variety of clinical settings for primarily hemostatic and surgical sealing applications. Examples in which fibrin-based materials can be implemented are cardiovascular surgery, neurosurgery, and thoracic surgery [174].

In this review, we have seen that fibrin can also be a very effective drug or cell carrier that can release these biological molecules or cells in a sustained manner to help build complicated wounds and to protect them against infection as well. Fibrin fibers feature unique mechanical properties that are closely associated with the wound healing process. This should not be overlooked in future studies, and methods to tune the mechanical properties of fibrin fibers should be studied in more detail. Nanofiber mat wound gauzes enriched with fibrin have been shown to be very effective against chronic wounds, but also for skin reconstruction. Biodegradable nanoparticles carrying fibrin can penetrate the skin and promote deep wound reconstruction. New techniques have been reported to engineer living bilayer human skin equivalent using human fibrin fibers. As such, it will soon be possible to construct a fully autologous human skin equivalent using culture-expanded human skin cells and fibrin fibers extracted from human blood [175,176,177,178]. We have also reviewed several studies in which, as cell-delivery vehicles, fibrin fibers were shown to facilitate cell attachment, growth, and differentiation and, ultimately, tissue formation and organization, because of their unique fibrilar structure in combination with other biomedical materials. Similar studies reviewed herein also demonstrated that fibrin may be used as a biomaterial to deliver genes in an efficient, cell-controlled, and spatially localized manner for potential applications in vitro or in vivo. When implanted in vivo, fibrin hydrogels were shown to enable human mesenchymal stem cells to migrate out of the fibrin gel and invade a calcium-carbonate-based ceramic scaffold, signifying that fibrin gels can serve as a delivery system for human mesenchymal stem cells.

We then focused our attention on drug-loaded fibrin-based materials and the mechanical properties of fibrin and fibrin containing wound repair structures. Several highly cited studies showed that fibrin can be used for the sustained delivery of drugs and bacteriophages; this strategy holds promise for many antibacterial applications. Antibiotics and several cancer drugs appear to be highly compatible with fibrin, with no loss in drug efficiency. Recent studies have also proven that drug-releasing fibrin structures and materials improve cell survival and differentiation, particularly when engineering tissue from stem cells. Furthermore, the controlled release of different drugs from fibrin-based hydrogels or films can enhance the survival of neural cells, as well as their differentiation into mature neural tissues. Fibrin-based scaffolds are hemostatic (inhibiting blood flow) and biodegradable in vivo and in vitro, and release matrices of small molecules at a controlled rate. Hence, they could be suitable candidates for drug delivery implants for short-term use. Long-term versions should be developed and tested in vivo as well. From the mechanical point of view relating to wound repair, the properties of individual fibrin fibers were shown to be determined by the number and packing arrangements of double-stranded half-staggered protofibrils. This is still not a completely understood phenomenon. It has also been proposed that the forced unfolding of sub-molecular structures, including the elongation of flexible and relatively unstructured portions of fibrin molecules, can contribute to fibrin deformation. The interplay between fibrin stiffness and elongation is not only an essential part of the biomechanics of hemostasis and thrombosis, but also a rapidly developing field of bioengineering that uses fibrin as a versatile biomaterial with exceptional and tunable biochemical and mechanical properties.

Future studies related to fibrin-based biomaterials should focus on fabrication techniques that would enable the manufacture of complex 3D structures with a defined geometry and architecture. In this way, it will be possible to assemble the physiological environment provided by hydrogels—for instance, into precise micro-scale structures—which is a major step towards the engineering of artificial 3D tissues and organs. Fibrin is a good starting point for creating extracellular matrices that can be easily approved for clinical applications. Pure fibrin hydrogels suffer from poor mechanical stability; therefore, modifications with additional proteins, polysaccharides, or synthetic polymers will lead to hydrogels with improved stability for various tissue regeneration procedures. Fibrinogen is usually extracted from biological sources; therefore, batch-to-batch variations will invariably appear that can hinder certain clinical requirements for a particular wound repair product. However, fibrinogen can be isolated from the milk of transgenic dairy cows or from yeast systems at higher production volumes with good repeatability. Very recently, Gaule and Ajjan [179] stressed that there are still issues related to the lack of agents that directly target the fibrinogen molecule. They further argued that designing Affimers (small proteins that bind to target proteins with affinity in the nano-molar range) can stabilize fibrin networks with the potential to control excessive bleeding. These engineered non-antibody-binding proteins, which are designed to mimic the molecular recognition characteristics of monoclonal antibodies in different applications and specifically bind fibrinogen and control clot stability/resistance to lysis, may prove to be clinically viable for the treatment of both bleeding and thrombotic disorders in wounds.

## Figures and Tables

**Table 1:** Summary of notable studies on skin reconstruction applications enabled by fibrin-based materials.

Fibrin-Based Material Description	Targeted Biomedical Application	Wound Type	Type of Cells Used	Animal Models	Clinical Application	Bio-Mechanical Tests	Comments	Reference
Pure fibrin gel	Skin substitute	Burn	Human keratinocytes	n/a	Yes	n/a	3-year patient follow up	[104]
Fibrin–succinimidyl glutarate blends	Skin-on-chip/skin reconstruction	Toxic wounds	Fibroblasts and keratinocytes	n/a	n/a	Permeation and swelling tests	Good potential for wound healing	[105]
Platelet-rich fibrin	Diabetic foot ulcers	Diabetic skin wound	n/a	Male nude mice	n/a	n/a	Promoting angiogenesis	[106]
Leukocyte- and platelet-rich fibrin	Scalp Defect Reconstruction	Surgical open wounds	n/a	n/a	Yes	n/a	Effective toward skin malignancy on the scalp	[107]
Collagen hydrogel/fibrin-coated polylactide	Skin repair	Deep skin wounds	Fibroblasts	n/a	n/a	n/a	Keratinocytes formed basal layers	[108]
Poly(ethylene glycol)–fibrinogen conjugates	Tissue engineering	n/a	Smooth muscle cells	n/a	n/a	Stress–sweep rheological testing	Cell proliferation control with fibrin nano-fibers	[109]
Fibrin hydrogel	Skin substitutes	Subcutaneous replacement	Adipose-derived stem cells (ASCs) and mature adipocytes	n/a	Patients undergoing body contouring surgery	n/a	Artificial hypodermis similar to native adipose tissue	[110]
Fibrin membrane	Skin scaffolds	Diabetic wound regeneration	Fibroblasts	Diabetic rats	n/a	n/a	Good collagen deposition in the wounds	[111]
Platelet-rich fibrin	Excisions of skin cancers	Dermatologic surgery	n/a	n/a	Patient with multiple nonmelanoma skin cancer.	n/a	Exuberant granulation tissue formation over ulcers	[112]
Leukocyte–Platelet-Rich Fibrin	Skull defect reconstruction	Endoscopic skull base surgery	n/a	n/a	Patients underwent endoscopic endonasal resection	n/a	Healthy crust formation occurred	[113]
Collagen–fibrin–polyethylene glycol (PEG) scaffolds	Vascular skin reconstruction	Burn-induced wound debridement	Stem cells from adipose tissue layer	Athymic rats	n/a	n/a	Effective against vascularized dermal equivalent for severe trauma cases	[114]
Fibrin/hyaluronan gels	Tracheal defects	Cartilage regeneration	Chondrocytes from rabbit	Female rabbits	n/a	n/a	No graft rejection recorded	[115]
3D fibrin constructs	Skin grafting	Dermo-epidermal skin substitutes	Adipose-derived cells	Immuno-incompetent female nu/nu rats	n/a	n/a	Successful prevascularizion of wound bed	[116]
Fibrin/hyaluronic acid (HA) hydrogel with poly(l-lactic-co-glycolic acid) (PLGA)	Reconstruction of trachea	Thyroid/laryngeal malignancies	Allogeneic chondrocytes	New Zealand white male rabbits	n/a	n/a	Successful neocartilage formation with minimal granulation tissue	[117]
Poly(l-lactide) modified fibrin	Ascorbic acid rich skin constructs	Heart valve	Human dermal fibroblasts	n/a	n/a	n/a	Promoted collagen production in the cells	[118]
Thrombin/fibrinogen embedded skin explants	Skin substitute	Skin explants in wound repair	Skeletal muscle explants	n/a	n/a	n/a	Excellent cell outgrowth from skin explants onto dermal substitute	[119]

**Table 2:** Drug encapsulating and sustained release systems based on fibrin and fibrin composite wound repair materials.

Fibrin-Based Material Description	Drug Inclusion	Targeted Application	Sustained Release Experiments	Animal Models	Clinical Application	Growth Factors/Cells	Reference
Poly(ether)urethane-polydimethylsiloxane/fibrin-based scaffold	Vascular endothelial growth factor (VEGF) and basic fibroblast growth factor (bFGF)	Diabetic skin ulcers	n/a	Male diabetic mice	n/a	Human growth factors	[127]
Hyaluronic acid–fibrin hydrogel	Dexamethasone and galectin-3 inhibitor	Inflammatory joint diseases	n/a	Rats (not specified)	n/a	n/a	[128]
Commercial fibrin sealant	Erythromycin and cefazolin	Postoperative antibiotic delivery	120 h release	n/a	n/a	n/a	[129]
Fibrin nanoparticles in chitosan	Ciprofloxacin and fluconazole	Polymicrobial wound infections	30 days release	Female SD rats and pig skin	n/a	n/a	[130]
PEGylated fibrin/chitosan gel	Silver sulfadiazine	Burn wounds	72 h release	n/a	n/a	n/a	[131]
Fibrin gel	Doxorubicin	Neuroblastoma	n/a	Female nude mice	n/a	Human LAN5, IMR32Luc+ and SHSY5YLuc+ cells	[132]
Fibrin nanoparticles	Ciprofloxacin and fluconazole	Diabetes therapy	150 h release	n/a	n/a	HDF Cell lines	[133]
Fibrin hydrogel	Cyclophosphamide	Unsatisfied cytoreductive surgery	100 h release	Female C57BL/6 and BALB/c mice	n/a	PD-L1 antibody and Cell line 4T1-luc	[134]
Fibrin gel	Plasminogen	Tympanic perforations	7-day release	Male diabetic mice	n/a	Mouse fibroblast cell line L929 and human keratinocytes HaCaT	[135]
Heparin-conjugated fibrin	Bone morphogenetic protein-2 (BMP-2)	Bone regeneration	30-day release	Sprague Dawley rats	n/a	Carvarial osteoblasts	[136]
Hollow fibrin microspheres	Human β nerve growth factor (NGF)	Neuronal dysfunctions	8-day release	Male Sprague Dawley rats	n/a	Rat mesenchymal stem cells	[137]
Fibrin–chitosan gel	Recombinant human epidermal growth factor (rhEGF)	General wound healing	14-day release	n/a	n/a	BALB/c 3T3 cells	[138]
Physiologically clotted fibrin	Gallic acid	Bone tissue engineering	80 h release	n/a	n/a	MG-63 cells	[139]
Autologous platelet-rich fibrin	Vancomycin	Bone tissue engineering	350 h release	n/a	n/a	n/a	[140]
Fibrin gel	Cisplatin and cisplatin–hyaluronate complexes	Tumor growth inhibition	70 h release	NOD-SCID mice	n/a	Murine B16 melanoma cells and human SK-Mel-28 melanoma cells	[141]
Fibrin nanoparticles	Metal nanoparticles	Not specified	n/a	Female balb/c mice	n/a	RAW 264.7 and NIH 3T3 cells	[142]
Freeze-dried fibrin	Arbekacin sulfate	Osteomyelitis	18-day release	Male outbred Wistar rats	n/a	n/a	[143]
Poly(lactic-co-glycolic acid) microparticles in fibrin glue	Bupivacaine	Postoperative pain	35-day release	Female Sprague Dawley rats	n/a	L929 mouse fibroblast cells	[144]
Liposomes/chitosan fibrin	Tirofiban	Antithrombosis	25-day release	n/a	n/a	n/a	[145]

**Table 3:** Mechanical properties of various protein fibers in comparison with fibrin. Compiled from Ref. [17].

Protein	Young’s Modulus (MPa)	Elongation before Break (%)
Fibrin fiber, non-cross-linked	1.0–2.0	226
Fibrin fiber, cross-linked	11.0–15.0	332
Elastin	1	150
Myofibrils	1	200
Resilin	1–2	190, 313
Fibronectin	0.1–3.5	700
Spider silk (Araneus Flag)	3	270
Fibrillin	0.2–100	>185
Intermediate filament	6–300	160–220
Mussel byssus	10–500	109
Collagen, tendon	160–7500	12
Microtubules	1000–1500	≤20
α-Keratin wet	2000	45
Actin	1800–2500	≤15
Collagen, cross-linked	5000–7000	12–16
Spider silk (Araneus MA)	10,000	27

## Data Availability

Not applicable.
